# Herbal Insights: Exploring the Therapeutic Potential of Indian Dietary Herbs in Diabetic Cardiomyopathy Management

**DOI:** 10.2174/0115733998315714240801193254

**Published:** 2024-08-09

**Authors:** Ritu Dahiya, Prabhnain Kaur, Vishal Kumar Vishwakarma, Aditya Singh, Ramesh K. Goyal

**Affiliations:** 1 Department of Pharmacology, Delhi Pharmaceutical Sciences and Research University, Delhi, India;; 2 All India Institute of Medical Sciences, Department of Pharmacology, New Delhi, Maharaja Bhagwan Baksh Sinh Nagar, Amethi, Sultanpur U. P., India;; 3 Faculty of Pharmacy, Integral University, Lucknow, India

**Keywords:** Dietary herbs, diabetic cardiomyopathy management, cardiac dysfunction, therapeutic strategies, holistic perspective, cardioprotective

## Abstract

**Background:**

Diabetic Cardiomyopathy (DCM) poses a substantial healthcare challenge, necessitating innovative therapeutic strategies. This review delves into the evolving role of traditional Indian dietary herbs in managing DCM, aiming to shed light on their potential contributions.

**Methods:**

A comprehensive examination of the existing body of literature was conducted, synthesizing data from studies exploring the effects of various Indian dietary herbs on DCM. Molecular mechanisms, clinical outcomes, and safety profiles were scrutinized to establish a holistic perspective on their therapeutic potential.

**Results:**

The review illuminates the multifaceted benefits of Indian dietary herbs in DCM management. These herbs have demonstrated efficacy in mitigating cardiac dysfunction, reducing oxidative stress, and modulating inflammatory responses. Molecular insights highlight their role in the intricate signaling pathways underlying DCM. Furthermore, their safety profiles render them promising candidates for adjunct therapy.

**Conclusion:**

Indian dietary herbs emerge as promising allies in the battle against DCM, offering a holistic approach to the management of this intricate condition. Their cardioprotective effects, coupled with their ability to address the underlying molecular mechanisms, herald a new era in DCM therapy. This review underscores the need for further research to harness the potential of these herbs fully and provides a beacon of hope for individuals affected by DCM.

## 1 INTRODUCTION

DCM represents a notable cardiovascular consequence associated with diabetes, presenting a significant concern within the healthcare field. Frequently overlooked amidst diabetes-related ailments like retinopathy and nephropathy, this condition stands out due to its impact on both the form and function of the heart. However, the continuous progression of dilated cardiomyopathy (DCM) and its asymptomatic beginning highlights the pressing need to discover innovative therapeutic approaches.

This review explores the realm of traditional Indian dietary herbs, which serve as a plentiful source of the therapeutic capabilities of nature. With a rich historical connection to traditional medical practices, these herbs present a promising opportunity for reshaping the field of DCM management. The medicinal potential of herbal traditions, which is sometimes undervalued or overestimated, warrants further investigation and encourages us to uncover the inherent wisdom that has been passed down through millennia.

The occurrence of diabetes has extended to a global level, giving rise to significant concerns over the associated complications, including diabetic cardiomyopathy (DCM). The significance of developing novel, secure, and effective treatments for diabetic cardiomyopathy (DCM) increases in tandem with the rising prevalence of diabetes. The significance of some herbs in alleviating cardiovascular disorders has been acknowledged by conventional systems of treatment similar to Ayurveda. It is, therefore, imperative to reexamine these historical therapies from a contemporary scientific perspective to comprehend and exploit their capacity in the management of DCM.

Our exploration leads us through a diverse range of Indian dietary herbs, each possessing distinct combinations of bioactive chemicals and medicinal attributes. The herbs discussed in this context possess various properties that are beneficial for heart health. Hawthorn is known for its relaxing effects on the heart, while cinnamon is recognized for its antioxidant capabilities. Together, these herbs offer a valuable collection of cardioprotective compounds. The diverse range of effects they exhibit, including anti-inflammatory, anti-fibrotic, vasodilatory, and lipid-lowering properties, align with the complex characteristics of dilated cardiomyopathy (DCM).

This review encompasses not just an examination of historical wisdom but also a meticulous evaluation of empirical data. In the exploration of herbal treatments, we meticulously examine the molecular pathways that underlie their therapeutic benefits. We also evaluate the clinical results by examining studies that have bravely questioned the conventional approach to managing dilated cardiomyopathy (DCM).

With the influence of fenugreek on glucose metabolism and the cardio-protective properties of garlic, these herbal remedies provide a glimmer of hope for individuals suffering from dilated cardiomyopathy (DCM). Complementary therapies should not be regarded as substitutes for traditional treatments but rather as valuable supplements that enhance the range of available treatment options for a problem that necessitates a holistic approach.

The objective of this study is to offer a thorough and informative resource for doctors, researchers, and individuals who are dealing with DCM. The exploration of the medicinal capabilities of Indian dietary herbs is founded upon historical knowledge, strengthened by contemporary scientific rigor, and motivated by the possibility of improved well-being in the future.

## 2 EPIDEMIOLOGY

The occurrence rates of varying degrees of cardiac failure in subjects with diabetes were found to range from 19% to 26% across many significant clinical trials [1, 2]. The current prevalence of diabetic cardiomyopathy (DCM) remains uncertain due to the limited availability of comprehensive data from diverse diabetic groups. Several research findings indicated that the incidence of diastolic dysfunction among individuals diagnosed with type 2 diabetes mellitus (T2DM) might be as high as 30% [3, 4]. Nevertheless, alternative investigations have documented a prevalence rate ranging from 40% to 60% [5, 6]. The limited sample sizes in these research works restrict their ability to accurately assess the actual frequency of a widespread condition, such as diabetes.

In a recent study, researchers examined the occurrence of heart failure (HF) and myocardial dysfunction (MD) in individuals diagnosed with type 1 diabetes mellitus (T1DM) who had been living with the condition for a minimum of 10 years. The study revealed a prevalence of 14.5% for MD and 3.7% for heart failure after a seven-year follow-up period [7]. There was a yearly incidence rate of 0.1% for MD and 0.02% for HF. Initially, it was noted that instances pertaining to heart failure (HF) were predominantly characterized by diastolic dysfunction, accounting for 85% of the total cases. The observed differences in the described prevalence of dilated cardiomyopathy (DCM) can perhaps be attributed to variations in patient selection criteria and the utilization of diverse imaging modalities for diagnostic purposes.

In a recent study involving large-scale randomized control trials (RCTs) on the efficacy of diabetic medications, it was found that approximately 8-10% of patients diagnosed with diabetes were admitted to hospitals due to HF. Subsequently, it has been observed that 50% of individuals diagnosed with a dysfunctional heart and a reduced left ventricular ejection fraction (HFrEF) also suffer from diabetes [8]. A separate RCT that specifically targeted patients with HF and retained left ventricular ejection fraction (LVEF) reported that 49% of the participants had diabetes [9].

In the cohort of individuals affected by diabetic cardiomyopathy, the observed cumulative mortality rate was 18% for 9 years. With a prevalence incidence of 1.1%, diabetic cardiomyopathy occurs very frequently in the wide population [10].

## 3 PATHOPHYSIOLOGY OF DCM

Diabetes profoundly impacts the cardiovascular system through three distinct mechanisms:

1. The development of CAD as a result of accelerated atherosclerosis.
2. The occurrence of cardiac autonomic neuropathy (CAN).
3. The manifestation of DCM.

Rubler *et al.* were the first to offer the initial documentation of DCM [11] in 1972. Myocardial dysfunction in people with diabetes without coronary artery disease (CAD), high blood pressure, or valvular heart disease (VHD) is the hallmark of *de novo* myocardial dysfunction (DCM) [11, 12]. The DCM is speculated to have multifactorial etiology. Insulin resistance, metabolic disruptions, microvascular illness, changes in the renin-angiotensin system (RAS), cardiac dysfunction, and myocardial fibrosis are among the hypothesised causes [13]. The pathophysiology of DCM is believed to be primarily influenced by chronic hyperglycaemia.

However, the progression of DCM entails complex pathways and the interplay of numerous metabolic and molecular processes occurring in the cardiac system and plasma.

The primary metabolic irregularities observed in individuals with diabetes include hyperglycemia, hyperlipidemia, and inflammation. The metabolic dysregulations associated with diabetes lead to the generation of reactive oxygen species (ROS) and nitrogen species, which leads to problems, such as diabetic nephropathy and cardiomyopathy [14, 15]. Heart failure and cardiac dysfunction result from a cascade of adaptive reactions triggered by these metabolic abnormalities, as demonstrated in Fig. (**1**).

## 4 POSSIBLE MECHANISM FOR THE ADVANCEMENT OF DCM

### 4.1 Lipotoxicity Occurring in the Myocardium

The build-up of triglycerides in adipocytes is mostly attributed to the heightened levels of circulating free fatty acids, which are raised as a result of diabetes and obesity. The deposition of ectopic fat in organs other than visceral and subcutaneous adipocytes leads to cellular and organ failure. This malfunction impacts multiple organs in the body, including skeletal muscle, the liver, pancreatic β cells, and the myocardium, mostly by hindering mitochondrial activity [16]. The term “lipotoxicity” is widely used to describe the phenomena being discussed. Glucose intolerance, high blood pressure, and abnormal cholesterol levels are observed as consequences of the fat buildup within the liver and skeletal muscle. This, in turn, leads to persistent inflammation and resistance to insulin.

Adipose tissue accumulation can be found around the heart and inside the myocardium. There are two different forms of pericardial fat: the outwardly located paracardial fat and the internally located epicardial fat near the epicardium. A study has found that individuals without cardiac disease but with a significant amount of epicardial fat, as assessed by computed tomography, are more likely to develop coronary artery disease (CAD) [17].

Enhanced concentrations of unbound fatty acids in the bloodstream result in a corresponding augmentation of fatty acid accumulation inside cardiomyocytes. Accumulation of excessive fatty acids within cells occurs in the form of lipid droplets and triglycerides. Simultaneously, there is an increase in diacylglycerol and ceramide, a kind of sphingolipid [18]. Diacylglycerol-induced activation of protein kinase C (PKC) exacerbates resistance to insulin and oxidative damage. In the human failing myocardium, it has been observed that there is an elevation in the concentration of diacylglycerol. This correlates with elevated PKC distribution in the cell membrane and reduced Akt functionality [19]. The aforementioned observations indicate that diacylglycerol serves as a detrimental lipid intermediary inside the cardiac system. Ceramide has been found to induce mitochondrial malfunction and promote the occurrence of oxidative stress. When cardiomyocytes are exposed to C6-ceramide, the activity of Akt is diminished, and the expression of brain natriuretic peptide (BNP) mRNA is enhanced. Furthermore, it has been demonstrated that the suppression of ceramide production in the cardiac muscle improves the state of lipotoxic cardiomyopathy [20]. These results indicate that the accumulation of ceramide contributes to the advancement of left ventricular hypertrophy and cardiac dysfunction. Existing research suggests that lipotoxicity primarily impairs diastolic function [21].

In diabetic cardiomyopathy, the excessive activation of peroxisome proliferator-activated receptor α (PPARα) due to an abundance of fatty acids leads to heightened fatty acid uptake through CD36, hence promoting the development of lipotoxicity. However, there have been findings suggesting a decline in the function of PPARα in hypertrophic and deteriorating hearts having no connection to diabetes. This drop causes a decline in the breakdown of fatty acids through β-oxidation, leading to an inadequate energy supply [22]. Given the lack of consensus surrounding heart failure as a whole, additional research is required to provide greater clarification on this matter.

### 4.2 Elevated Levels of Oxidative Stress in the Myocardium

The mechanistic contribution of diabetes to increased oxidative stress can be explained as follows: oxidative stress happens as a result of the dysregulation of the electron transport chain in mitochondria. Furthermore, elevated activity of the nicotinamide adenine dinucleotide phosphate (NADPH) oxidase and the renin-angiotensin system (RAS) exacerbates oxidative stress. Lastly, advanced glycation end products (AGEs) lead to an escalation of oxidative stress. Advanced glycation end products (AGEs) have been observed to induce not only direct cellular damage but also to elevate oxidative stress levels by promoting reactive oxygen species (ROS) generation [23]. The activation of NADPH oxidase and consequent elevation in oxidative damage and irritation are enabled by the interaction between secreted extracellular advanced glycation end products (AGEs) and cell surface receptors called receptors for advanced glycation end products (RAGE). Redox imbalance has been found to cause cardiac fibrosis and hypertrophy [23–25].

Previous studies demonstrated that elevated glucose levels could impede the antioxidant signalling pathways mediated by Nrf2 and Sirt1 while simultaneously activating the inflammatory signalling pathway mediated by NF-κB [26]. The interplay between inflammatory processes and oxidative stress results in the elevation of inflammatory mediators and reactive oxygen species (ROS), hence facilitating and intensifying heart failure and myocardium remodelling.

H_2_S (Hydrogen Sulfide), a potent gasotransmitter, potentially holds significant implications within the cardiovascular system. It was observed that the hearts of mice suffered from severe diabetic cardiomyopathy due to a shortage in hydrogen sulfide (H_2_S), which further escalated mitochondrial damage, enhanced the buildup of ROS, facilitated necroptosis, and triggered activation of the NLRP3 inflammasome [27].

### 4.3 Mitochondrial Dysfunction

Insulin resistance is correlated with diminished glucose utilization and impaired oxidative metabolism, leading to an unbalanced process of fatty acid absorption and breakdown. Ultimately, these factors culminate in the occurrence of mitochondrial malfunction [28]. Moreover, observations have shown that in diabetic patients, there is a gradual decline in mitochondrial function inside cardiomyocytes. This decline leads to the buildup of lipids and subsequently triggers the production of a significant quantity of ROS. The presence of ROS exacerbates oxidative stress, hence exacerbating the condition of diabetic cardiomyopathy and further compromising the overall functioning of the myocardium [29].

Mitophagy, a type of autophagy, occurs within dysfunctional mitochondria, playing a crucial role in maintaining mitochondrial integrity. The process of mitophagy serves as a preventive mechanism in the context of diabetic cardiomyopathy, primarily by eliminating dysfunctional mitochondria. This clearance of aberrant mitochondria effectively mitigates oxidative stress and subsequently decreases cardiac apoptosis [28]. Nevertheless, an excessive amount of mitophagy has the potential to worsen cardiac damage in individuals diagnosed with diabetic cardiomyopathy. Multiple signalling mechanisms that govern the process of mitophagy have been found, such as the PINK1/parkin, AMPK-mTOR, and Wnt pathways.

### 4.4 Inflammation

**Fig. (2):**
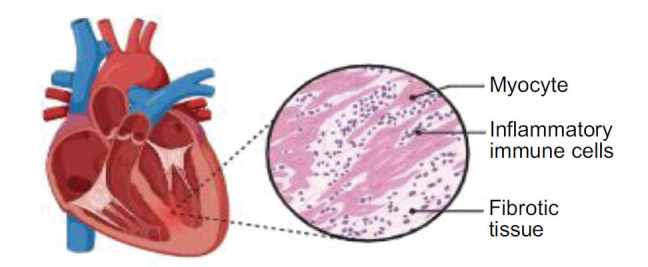
Inflammation of heart muscles.

The presence of diabetes leads to a chronic state of inflammation that is enhanced by increased inflammasomal activation. The NLRP3 inflammasome, a member of the “NOD-like” (NLR) receptor proteins, is characterised by the presence of the pyrin domain and has been linked with the advancement of diabetic cardiomyopathy [30, 31]. The activation of NLRP3 is induced by raised amount of free fatty acids (FFA), poor insulin metabolic signalling, and increased blood glucose concentrations. The stimulation of the NLRP3 protein complex induces the generation of interleukin-1 beta (IL-1β) and interleukin-18 (IL-18), hence initiating localised inflammation in the surrounding tissue. Nuclear factor-kB (NF-kB) and thioredoxin-interacting/-inhibiting protein (TXNIP) promote the activation of caspase-1 and IL-1beta, acting as the effectors of the NLRP3 inflammasome in response to ROS [31]. The myocardium of rats with diabetes exhibited the incidence of pyroptosis mediated by the NLRP3 inflammasome, which is a regulated type of necrotic cell death [32]. The previous work indicated that the therapeutic intervention involving the suppression of the NLRP3 gene has positive outcomes in terms of cardiac inflammation, necrosis, fibrosis, and cardiac dysfunction (Fig **2**).

### 4.5 Inappropriate Calcium Handling in the Heart

The presence of Type II diabetes in mice has been observed to result in compromised contractility and relaxation. This can be attributed to enhanced intracellular resting calcium levels, a deceleration of calcium transients, a decrease in the calcium pumping process conducted by the sarcoplasmic reticulum, and a hindered absorption of calcium by the sarcoplasmic reticulum [33, 34].

### 4.6 Disruptions to Cardiac Autonomic Function

Cardiac autonomic imbalance, often known as neuropathy, encompasses deviations in the regulation of cardiac rhythm, circulatory dynamics, and cardiac anatomy and physiology [35]. Hyperglycaemia is a significant contributing factor that accompanies the occurrence of redox imbalance and the formation of toxic glycosylation products. These processes subsequently give rise to neuronal dysfunction and, ultimately, neuronal death. Fig. (**3**) illustrates the possible mechanisms responsible for the DCM.

## 5 OTHER MECHANISMS

Heart failure is a multifaceted condition that can be further exacerbated by the presence of diabetes. In this context, there are additional pathogenic mechanisms that accord for the progression and advancement of heart failure. These mechanisms include salt retention resulting from hyperinsulinemia, as well as impaired function of vascular endothelium. The vascular endothelium plays a crucial role in regulating diverse physiological processes, encompassing the modulation of contraction and relaxation of the vascular wall, the facilitation of adhesion of inflammatory cells to the vascular wall, controlling vascular permeability, and regulating the coagulation and fibrinolytic system. One of the noteworthy chemicals in this particular situation is nitric oxide (NO), which is synthesised by endothelial nitric oxide synthase (eNOS). The vasodilatory effects of nitric oxide are well-documented since it has been shown to cause relaxation in vascular smooth muscle. This physiological response is of great significance in the modulation of cardiac tone.

Individuals suffering from cardiac insufficiency have endothelial dysfunction [36], which has been linked to a heightened danger of death in individuals with both ischemic and non-ischaemic heart failure [37]. Vascular endothelial dysfunction is observed in the first stages of reduced glucose tolerance in patients with T2DM, and it upholds a significant role in the advancement and succession of arteriosclerosis and coronary artery disease. Several acknowledged factors contribute to vascular endothelial dysfunction. These factors are comprised of hyperglycaemia, insulin resistance, low blood sugar, and postprandial hyperlipidaemia [38, 39].

Under diabetes circumstances, there is an elevation in the absorption of glucose inside the endothelial cells through the glucose transporter 1 (GLUT 1). Subsequently, the signalling of protein kinase C (PKC) and the stimulation of advanced glycation end products (AGEs) are augmented, leading to disturbances in intracellular metabolism and impairment of endothelial cell function. The phosphoinositide 3-kinase/Akt (PI3K/Akt) pathway, a constituent of the insulin signalling path, exerts nitric oxide (NO)-mediated vasodilatory effects on endothelial cells of the vascular system. Insulin resistance results in compromised signalling within this system, resulting in the gradual impairment of the function of vascular endothelial cells [40, 41]. It is widely believed that hypoglycemia leads to vascular endothelial dysfunction due to increased levels of reactive oxygen species and catecholamines [42], as well as an upregulation of inflammatory cytokines [43]. Instead of a chronic rise in blood glucose levels, the occurrence of a rapid spike in blood glucose following a meal, known as postprandial hyperglycemia, specifically glycemic fluctuation, has been found to augment the synthesis of inflammatory cytokines and induction of oxidative stress, resulting in a declined vascular endothelial function.

The elevation of blood triglyceride (TG)-rich lipoprotein concentration is attributed to two factors: increased presence of chylomicron remnants resulting from dietary influences (extrinsic) and heightened production of very low-density lipoproteins (VLDL) inside the hepatic system (intrinsic). Moreover, the prolonged dispensation of triglyceride-rich lipoprotein leads to the development of non-fasting hypertriglyceridemia, also known as non-fasting hyperlipidaemia. Obese individuals, specifically, exhibit hypertriglyceridemia following meals at an early stage, whereas those with insulin resistance, commonly referred to as metabolic syndrome patients, experience chronic elevated levels of triglycerides. The impairment of vascular endothelial function is observed in individuals with non-fasting hyperlipidaemia [39]. It is believed that the elevated levels of chylomicrons and VLDL remnants stimulate the synthesis of inflammatory cytokines and free radicals, leading to a reduction in eNOS activity and vascular endothelial function [39, 44]. Ingesting a diet rich in fats leads to an elevation in blood levels of triglycerides [TG], apolipoprotein B-48 (ApoB-48), and cholesterol associated with remnant-like lipoproteins. These levels reach their highest point approximately four hours after the meal. It is noteworthy that there is a drop in postprandial brachial artery flow-mediated dilation (FMD), and this decrease reaches its lowest level in the fourth hour following the consumption of an oral fat load. Additionally, a decline in vascular endothelial function was also found [39].

## 6 BIOMARKERS IN DCM

Biomarkers are crucial in diagnosing, predicting outcomes, and monitoring diabetic cardiomyopathy (DCM). Various biomarkers have been discovered to offer insights into the underlying mechanisms and advancement of this condition. Here are some key biomarkers associated with DCM:

### 6.1 Natriuretic Peptides (BNP and NT-proBNP)

B-type natriuretic peptide (BNP) and its inactive N-terminal prohormone (NT-proBNP) are well-known biomarkers for heart failure.

Elevated levels of BNP and NT-proBNP in individuals with diabetes may indicate the presence of diabetic cardiomyopathy and an increased likelihood of developing heart failure [5].

The main energy source for cardiac contractility is derived from the oxidation of fatty acids within mitochondria, which are plentiful in the myocardium. However, a variety of energy sources, such as carbohydrates, lipids, amino acids, and ketone bodies, are utilized by the heart. In persons with type 2 diabetes mellitus (T2DM), insulin resistance impedes glucose entry into cardiomyocytes, leading to increased fatty acid oxidation, lipid accumulation, and consumption of oxygen. This ultimately compromises cardiac function.

During cardiac stress and various systemic pathological conditions, there is an excessive generation of atrial natriuretic peptide (ANP) and B-type natriuretic peptide (BNP) as a compensatory mechanism. Elevated concentrations of NPs in T2DM patients serve as a warning sign of cardiovascular instability. Monitoring high NP levels is crucial in identifying potential cardiovascular complications, particularly in the context of type 2 diabetes.

Higher levels of NT-proBNP have emerged as a discriminator, indicating an augmented risk for cardiovascular disease (CVD) in patients with type 2 diabetes mellitus (T2DM) and microalbuminuria, as evidenced in the Steno-2 study. Specifically, NT-proBNP levels exceeding the median are linked with an elevated risk of adverse cardiovascular events. In the same study, a noteworthy finding indicated that a 10 pg/mL reduction in plasma NT-proBNP over an initial 2 years of intervention was associated with a 1% relative decline in the primary composite outcomes, including cardiovascular mortality, myocardial infarction (MI), revascularization procedures, stroke, and amputations [45].

Furthermore, the integration of NT-proBNP into multivariable models significantly enhanced the predictive accuracy for cardiovascular outcomes in distinct cohorts. For instance, in the Sun-MACRO study involving patients with T2DM and macroalbuminuria, incorporating NT-proBNP enhanced the prediction of cardiovascular events [46]. Similarly, in the TREAT trial involving T2DM patients having anaemia and predialysis chronic kidney disease, the incorporation of NT-proBNP along with troponin T demonstrated a substantial enhancement of 17.8% in predicting cardiovascular outcomes [47].

NT-proBNP levels equal to or greater than 45.2 ng/L and CAC levels exceeding 400 are equally strong predictors of composite cardiovascular endpoints and mortality. Notably, while CAC exhibited a correlation with atherosclerotic cardiovascular disease (ASCVD), levels of NT-proBNP showed a robust correlation with cardiac dysfunction and possibly other changes in the circulatory system. [48].

Tarnow *et al.* found that improved levels of NT-proBNP in the bloodstream were indicative of higher rates of both general mortality and mortality related to cardiovascular issues in patients with type 2 diabetes mellitus, including those who did not have any prior cardiovascular diseases [49]. Additionally, Bruno *et al.* affirmed NT-proBNP as an independent predictor of cardiovascular mortality risk in aging individuals with type 2 diabetes, irrespective of earlier CVD implicated [50].

New findings, as observed in the analysis of the ELIXA trial, reinforce the singular predictive efficacy of NP. In this context, NT-proBNP alone exhibited predictive accuracy comparable to that of an evident multivariable model for mortality in patients with T2DM enrolled within 180 days following an acute coronary syndrome (ACS) [51]. These findings collectively underscore the significant role of NT-proBNP as a robust biomarker in predicting severe cardiovascular events and mortality in patients with evident type 2 diabetes.

### 6.2 Cardiac Troponin

Cardiac troponins (cTnT and cTnI) are well-known indicators of myocardial damage and are commonly utilized in the diagnosis of acute coronary syndromes.

Elevated troponin levels have been pragmatic in patients with diabetic cardiomyopathy, indicating myocardial damage or stress [23].

Elevated cardiac troponin levels can signify myocardial damage, even when acute coronary events are absent. In DCM, troponin elevation may indicate ongoing myocardial damage or stress, contributing to the diagnostic evaluation of cardiac involvement in diabetes.

Troponin levels in diabetic patients may serve as valuable indicators for risk stratification. Higher troponin concentrations have been linked with adverse cardiovascular outcomes in individuals with diabetes, providing important prognostic information.

Monitoring troponin levels in diabetic patients can aid in the early detection of cardiac complications, prompting timely intervention and appropriate management strategies. This is particularly crucial as DCM may progress silently, and troponin assessment can contribute to a more comprehensive approach to cardiovascular care in diabetes.

The study referenced emphasizes the functions of troponins in the diagnosis, risk assessment, and treatment of hypertensive and diseased diabetic heart conditions. It recognizes troponin elevation as a marker of myocardial involvement in these conditions, reflecting the broader impact of diabetes on cardiac health [52].

### 6.3 Inflammatory Markers

Inflammation plays a critical role in the advancement and escalation of diabetic cardiomyopathy.

Biomarkers like interleukin-6 (IL-6), C-reactive protein (CRP), and tumor necrosis factor-alpha (TNF-α) are related to inflammation and may serve as indicators of cardiac inflammation in diabetes [53].

C-reactive protein (CRP), a component of the pentraxin superfamily, is widely recognized as an indicator of acute systemic inflammation [54]. CRP, when evaluated with extremely sensitive assays, is a powerful biomarker for determining the risk of cardiovascular disease (CVD). It can even be effective at detecting low quantities of CRP, which is now referred to as high-sensitivity CRP [hs-CRP] [55].

The hs-CRP levels in serum have demonstrated independent predictive capability for the initiation of type 2 diabetes (T2DM) and cardiovascular disease in the general population [56, 57]. Elevated levels of hs-CRP have been accompanied by T2DM and various indicators of diabetes-related cardiovascular risk [58, 59]. Notably, Aryan Z. *et al.* (2018) [60], in a study based on a certain population, established that hs-CRP anticipated diabetic kidney disease, heart failure, and its associated cardiovascular complexities in overt T2DM patients. Moreover, the research showcased a notable improvement in the differentiating capability of standard cardiovascular risk evaluation tools with the addition of hs-CRP [60].

In individuals with established atherosclerotic CVD, heightened hs-CRP levels have been linked to unfavourable results of patients, particularly among non-T2DM patients [61, 62]. Furthermore, Yang QQ *et al.* [20^20^] [63] provided an evident finding claiming a direct correlation between levels of hs-CRP and the severity of major depressive disorder. Reductions in levels of hs-CRP amid dyslipidemia or coronary artery disease therapy have shown close associations with improved long-term prognoses [64]. It is noteworthy that despite nearly 40% of HFpEF patients exhibiting typical hs-CRP values, those with reduced ejection fraction (HFrEF) displayed higher hs-CRP levels, predicting myocardial impairment and HFrEF post-acute myocardial infarction [65–67]. Unexpectedly, a decline in hs-CRP levels during acute heart failure was associated with a significant decrease in the 3-year mortality risk among patients with heart failure with preserved ejection fraction (HFpEF) but not among those with heart failure with reduced ejection fraction (HFrEF) [68].

In summary, despite the recognized association of hs-CRP with clinical events and its status as a predictor that stands alone in forecasting cardiovascular morbidity and mortality [68], uncertainties persist regarding its predictive ability in patients with different heart failure phenotypes, leading to considerable heterogeneity in present cardiovascular risk factors.

### 6.4 Fibrosis Markers

Fibrosis is a hallmark of diabetic cardiomyopathy, leading to impaired myocardial function.

Biomarkers like galectin-3, transforming growth factor-beta (TGF-β), and procollagen type III amino-terminal peptide (PIIINP) are associated with fibrosis and extracellular matrix remodelling [69].

Galectin-3, belonging to the galectin family, is a lectin that binds to β-galactosidase, exhibiting a range of biological functions encompassing cell growth modulation, differentiation, proliferation, pre-mRNA splicing, apoptosis suppression, angiogenesis attenuation, reduction in inflammation, and fibrosis [70-73]. Its deficiency is associated with tissue injury, while over-expression is linked to tissue protection. Galectin-3 represents a promising cardiac biomarker for prognosis, holding therapeutic promise [74].

The Dallas Heart Study indicated a link between galectin-3 levels and both the occurrence and development of type 2 diabetes (T2DM), as well as various fat compartments [75, 76]. Elevated serum galectin-3 levels are independently associated with advancing renal disease in T2DM, cardiovascular disease (CVD), and a heightened risk of all-cause mortality [75, 76].

Elevated levels of galectin-3 in the bloodstream have been correlated with heart failure (HF), negative cardiac remodeling, myocardial fibrosis, atrial fibrillation, and coronary artery disease (CAD). Galectin-3 is indeed an influential prognostic biomarker for HF and a moderator of T2DM advancement [77, 78]. Among individuals diagnosed with heart failure and preserved ejection fraction (HFpEF), galectin-3 levels are positively linked to several factors, such as age, creatinine clearance, arterial stiffness, aldosterone, and B-type natriuretic peptide (BNP) levels. There is a negative correlation between galectin-3 levels and left ventricular ejection fraction [79-81]. In the DIAST-CHF study, galectin-3 levels exceeding 313.57 ng/mL were found to predict incident HFpEF, adjusted all-cause mortality, and the adjusted composite of cardiovascular hospitalization and death [82]. A positive relationship between levels of galectin-3 and the severity of heart failure was determined by Gocer H. *et al.* (2019), whereas they found a negative relationship between galectin-3 levels and left ventricular ejection fraction [83].

Increased levels of galectin-3 were observed to be a strong interpreter of death in cases of acute heart failure, including both forms of chronic heart failure [84]. Despite recent developments demonstrating that galectin-3 is not much effective as other biomarkers in predicting mortality, the combination of galectin-3 and N-terminal pro-B-type natriuretic peptide (NT-proBNP) has demonstrated an enhanced ability to accurately predict mortality compared to each biomarker used alone [85, 86]. In general, galectin-3 has shown promise in predicting heart failure beyond traditional biomarkers like NPs and cardiac troponins. However, its effectiveness in the point-of-care management of heart failure patients needs more exploration in upcoming research.

### 6.5 Advanced Glycation End Products (AGEs)

In diabetes, high blood sugar levels lead to the creation of advanced glycation end products (AGEs), which cause damage to both the blood vessels and heart muscles.

Elevated levels of circulating AGEs may serve as biomarkers reflecting the cumulative glycaemic exposure and oxidative stress associated with diabetic cardiomyopathy [87].

### 6.6 Markers of Oxidative Stress

Oxidative stress is implicated in the development of diabetic complications, including cardiomyopathy.

Biomarkers like8-hydroxy-2'-deoxyguanosine (8-OHdG), malondialdehyde (MDA), and superoxide dismutase (SOD) activity may indicate increased oxidative stress in the myocardium [88].

### 6.7 MicroRNAs (miRNAs)

MicroRNAs, small non-coding RNAs involved in gene regulation, play a significant role. Certain miRNAs like miR-1, miR-133a, and miR-208a are known to be involved in the regulation of cardiac function and are found to be altered in cases of diabetic cardiomyopathy [89].

### 6.8 Myocardial Imaging Biomarkers

Imaging biomarkers, like cardiac magnetic resonance imaging (MRI) or echocardiography-derived measures, can provide structural and functional insights into the myocardium in diabetic cardiomyopathy [90].

Understanding the dynamics of these biomarkers in the context of diabetic cardiomyopathy may not only aid in early detection but also guide therapeutic interventions and monitor disease progression. However, it is essential to note that additional research is necessary to validate these biomarkers and establish their clinical utility in routine practice. Additionally, the integration of multiple biomarkers in a panel may offer a more comprehensive assessment of diabetic cardiomyopathy.

## 7 CURRENT TREATMENT FOR DCM

The management of heart failure is analogous among individuals with and without diabetes. Nonetheless, it is imperative to prioritise the administration of antidiabetic medications that are both safe and effective in reducing heart failure (HF)-related events, as these treatments can have varying effects on individuals with HF [91]. This review provides a description of the effects of several medicines. Diabetic cardiomyopathy represents a distinct condition. However, the pharmaceutical agents mentioned subsequently may or may not exert influences on various cardiovascular outcomes in individuals with diabetes.

The presence of a U-curve phenomenon has been observed in the correlation between HbA1c levels and mortality among individuals with both heart failure and diabetes [92]. Consequently, achieving a favourable prognosis through the exclusive means of reducing blood glucose levels proves to be challenging. Furthermore, it is postulated that hypoglycemia exerts harmful effects on the cardiovascular system by eliciting sympathetic nerve activity and inflammation. Consequently, it is imperative to prevent the incidence of hypoglycemia.

Several dissenting perspectives exist regarding the effectiveness of strict glycemic control in preventing heart failure initially. Results from a meta-analysis comparing intensive glycemic control to conventional methods showed no significant decrease in the likelihood of hospitalization specifically linked to heart failure with intensive therapy [93]. Furthermore, it has been noted that the implementation of rigorous glucose-lowering therapy could potentially worsen heart failure. A comprehensive meta-analysis comprising 13 trials involving a total of 34,533 individuals diagnosed with type 2 diabetes mellitus (T2DM) revealed that the implementation of rigorous glucose-lowering treatment did not yield a decrease in cardiovascular events. Conversely, this approach was found to elevate the likelihood of developing heart failure by 47% [94]. Nevertheless, there is currently no pharmacological intervention specifically designed for the management of dilated cardiomyopathy (DCM). Hence, it is imperative to investigate compounds that possess the ability to selectively address or control dilated cardiomyopathy (DCM). One potential avenue for investigating these molecules is in the plants documented in traditional and complementary medicine literature. These herbs can act as promising initial options for the synthesis and expansion of more sophisticated pharmaceutical compounds.

## 8 INDIAN HERBS IN DCM

**Fig (4):**
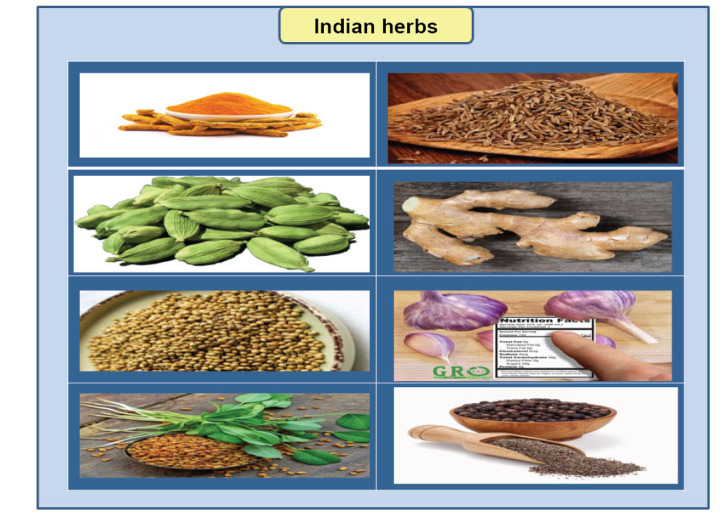
Indian herbs in the management of DCM.

The management of diabetic cardiomyopathy often entails traditional medical interventions, including lifestyle adjustments and the administration of prescribed drugs. However, certain individuals may also consider complementary and alternative methods, such as herbal medicines, as additional or supportive treatments [95]. Below are several herbal remedies that have been extensively researched in relation to diabetes and cardiovascular health, specifically focusing on diabetic cardiomyopathy (Fig. **4**).

### 8.1 Cinnamon (*Cinnamomum Cassia*)

Several studies have indicated that cinnamon may enhance blood sugar regulation in individuals with diabetes. Although it may have a beneficial influence on glycemic management, its exact impact on diabetic cardiomyopathy is uncertain and necessitates additional investigation [96].

### 8.2 Bitter Melon (*Momordica Charantia*)

Bitter melon is a traditional remedy used in some cultures to manage diabetes. The findings of one of the studies indicate that while BM extract may not be suitable as a standalone treatment, it has the potential to greatly improve the efficacy of medications currently used to treat obesity-related conditions, such as type 2 diabetes and cardiovascular illnesses. The utilisation of this plant as a supplementary therapy is anticipated to be highly beneficial in reducing the amount of damaged cardiac tissue caused by infarction and enhancing blood flow after an ischemia-reperfusion insult to the organ [97].

### 8.3 Fenugreek (*Trigonella Foenum-Graecum*)

Fenugreek seeds contain groups that may help to enhance insulin sensitivity and reduce blood sugar levels. Several studies provide evidence that fenugreek seed extract has significant therapeutic potential in the treatment of dilated cardiomyopathy and other cardiovascular illnesses. This potential is attributed to its ability to improve metabolic imbalances and reduce oxidative stress, as well as regulate genes associated with apoptosis [98].

### 8.4 Hawthorn (*Crataegus*)

Hawthorn is often used in traditional herbal medicine to support heart health. In a specific study, the level of SOD activity in rats of the DCM group exhibited a significant drop, whereas the content of MDA showed a considerable increase in contrast to the results obtained for the control group. Remarkably, following HLF treatment, the enzymatic activity of SOD exhibited a rise, while the concentration of MDA experienced a notable drop. This study provided evidence that HLF has antioxidant properties [99].

### 8.5 Ginger (*Zingiber officinale*)

Ginger exhibits anti-inflammatory properties and might aid in regulating blood sugar levels. Ginger extract treatment decreased cardiac fibrosis and inflammation during diabetic cardiomyopathy, potentially by modulating the gene expression implicated in the SMAD/TGF-β pathway [100].

### 8.6 Garlic (*Allium sativum*)

Garlic has been studied for its potential cardiovascular benefits, such as blood pressure reduction and cholesterol management. The potential benefit of nano-curcumin and AGE suspension in treating DCM lies in their ability to reduce myocardial fibrosis, cardiac inflammation, and automated myocardial cell death. This is achieved by decreasing oxidative stress and the buildup of advanced glycation end products in the diabetic heart tissue [101].

### 8.7 Turmeric (*Curcuma longa*)

Turmeric comprises an active ingredient called curcumin, which possesses antioxidant and anti-inflammatory properties. Curcumin has the ability to hinder the generation of reactive oxygen species, diminish cardiac apoptosis, and decrease myocardial lipid buildup [102].

### 8.8 Bilberry (*Vaccinium myrtillus*)

Bilberry, related to blueberries, contains antioxidants that may be beneficial for cardiovascular health. The advantages of incorporating bilberry supplementation into a nutritious diet are substantiated by mechanistic investigations, animal research, and certain clinical trials [103].

Ayurveda, the traditional medicine of India, uses many herbs that have been suggested to offer potential advantages in managing diabetes and related consequences, such as diabetic cardiomyopathy. Table **1** indicates some Indian herbs that have been extensively explored for their potential therapeutic effects in the treatment of cardiomyopathy induced by diabetes.

## 9 FLAVONOIDS IN THE MANAGEMENT OF DIABETIC CARDIOMYOPATHY

Flavonoids belong to a category of natural compounds that are found abundantly in various plant-based foods and have been extensively researched for their unexplored health benefits, including their role in managing diabetes and diabetic cardiomyopathy. Diabetic cardiomyopathy involves structural and functional alterations in the heart that occur as a result of diabetes.

Table **2** demonstrates some flavonoids that have been studied for their potential effects in managing diabetes-induced cardiomyopathy:

## 10 DISCUSSION

The exploration of Indian dietary herbs and their potential in the management of diabetic cardiomyopathy has been a thought-provoking endeavor deserving of meticulous contemplation. As the discourse surrounding these findings unfolds, several crucial aspects emerge as prominent.

1. The therapeutic potential of Indian dietary herbs in the management of DCM is emphasised by their diverse range of activities. Fenugreek, garlic, and cinnamon, along with other substances, have demonstrated the capacity to alleviate the symptoms of diabetic cardiomyopathy and exert a significant influence on the fundamental pathophysiological mechanisms involved. The potential therapeutic value of these substances lies in their ability to reduce inflammation, counteract oxidative stress, and modulate biological pathways, making them promising candidates for comprehensive management of DCM.
2. Integrative Approaches: Although these herbs show potential, it is crucial to underscore their function as supplementary therapies rather than independent remedies. The use of herbal medicines in comprehensive strategies for managing DCM, in conjunction with known pharmaceutical and lifestyle therapies, has the potential to provide a more well-rounded and efficacious strategy.
3. In addition to mitigating DCM, the herbs addressed in this context may confer supplementary health advantages, contributing to holistic well-being. For example, the capacity of garlic to reduce blood pressure and the glucose-modulating actions of cinnamon contribute to the overall state of well-being, aligning with the holistic concepts observed in traditional medicine.

## CONCLUSION

Indian dietary herbs offer a compelling natural approach to controlling DCM, adding an exciting element to the effort of enhancing cardiovascular health. The amalgamation of conventional wisdom and modern scientific scrutiny propels us toward a more auspicious future in the management of DCM. The medical potential of Indian dietary herbs shows great promise, but fully realising their potential requires continuous inquiry, evidence-based practice, and collaborative efforts.

Examining the coexistence of historical and modern elements, along with the interaction between traditional remedies and current scientific advancements, offers a chance to investigate a holistic strategy for managing DCM. The purpose of our voyage is to investigate the extensive knowledge of herbal medicines in order to improve cardiovascular health, encourage a more positive future, and develop a deeper awareness of the healing potential present in the natural world.

## Figures and Tables

**Fig. (1):**
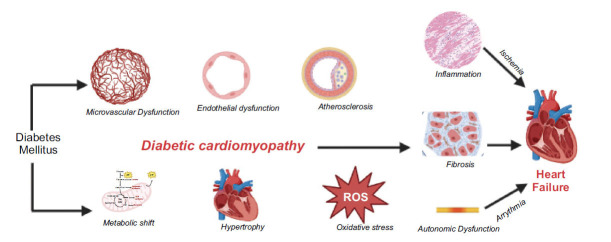
Pathophysiology of cardiomyopathy associated with diabetes.

**Fig. (3):**
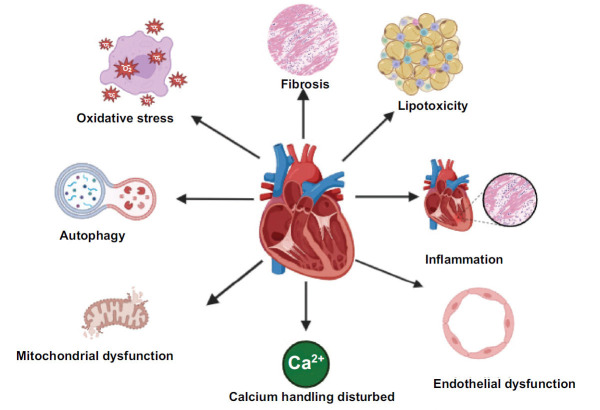
Possible mechanisms for the pathophysiology of DCM.

**Table 1:** Major Indian herbs in the management of diabetes-induced cardiomyopathy.

Herb	Scientific Name	Potential Benefits in DCM	References
**Bitter Melon**	*Momordica charantia*	Improves blood sugar control, which may indirectly benefit the heart.	[97]
**Cinnamon**	*Cinnamomum verum, others*	Potential anti-inflammatory and antioxidant properties.	[96]
**Gymnema Sylvestre**	*Gymnemasylvestre*	Helps regulate blood sugar levels and reduces oxidative stress in the heart.	[104]
**Fenugreek**	*Trigonella foenum-graecum*	Improves glycemic control and has antioxidant effects on the heart.	[98]
**Terminalia Arjuna**	*Terminalia arjuna*	Cardioprotective effects.	[105]
**Turmeric**	*Curcuma longa*	Antioxidant and anti-inflammatory properties that may be advantageous for the heart.	[102]
**Black Pepper**	*Piper nigrum L.*	Managing inflammation helps reduce arterial damage and the progression of atherosclerosis.	[106]
**Cardamom**	*Elettaria cardamomum.*	Boosted antioxidant enzyme production and activity in diabetes, reducing oxidative stress and inflammation.	[107]
**Clove**	*Syzygium aromaticum*	Reduced total cholesterol, LDL cholesterol, VLDL cholesterol, triglycerides.	[108]
**Curry leaves**	*Murraya koenigii.*	Anti-inflammatory, anti-hypertensive, and lowers total cholesterol.	[109]
**Black Cumin**	*Nigella sativa*	Antioxidant, anti-inflammatory, bronchodilatory, antihistaminic, anti-leukotriene, antifibrotic, and immunomodulatory properties.	[110]
**Fennel seeds**	*Foeniculum vulgare.*	Anti-oxidant, anti-thrombotic, and anti-inflammatory.	[111]

**Table 2:** Flavonoids in the treatment of diabetes-induced cardiomyopathy.

**Flavonoid**	**Structure**	**Food Sources**	**Potential Benefits in Diabetic Cardiomyopathy**	**References**
Quercetin	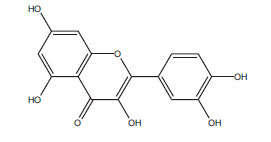	Onions, apples, tea, grapes	Reduce inflammation and oxidative stress in the heart.	[112]
Epicatechin	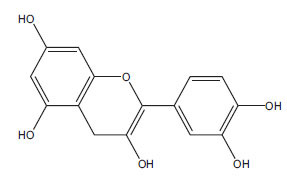	Dark chocolate, green tea	Antioxidant and anti-inflammatory properties	[113]
Resveratrol	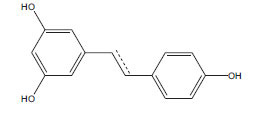	Red grapes, red wine	Improve cardiac function, reduce inflammation, and protect against oxidative damage.	[114]
Luteolin	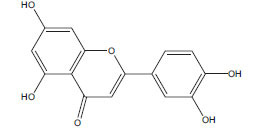	Celery, parsley, broccoli	Anti-inflammatory and antioxidant properties that could benefit heart health.	[115]
Kaempferol	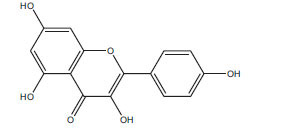	Kale, spinach, broccoli	Reduce oxidative stress and inflammation in the heart.	[116]
Myricetin	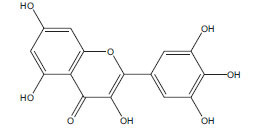	Berries, nuts, and red wine	Have antioxidant properties and protect the heart from oxidative damage.	[117]

## LIST OF ABBREVIATIONS

DCM: Diabetic Cardiomyopathy
CAD: Coronary Artery Disease
VHD: Valvular Heart Disease
LVEF: Ventricular Ejection Fraction
RAS: Renin-Angiotensin System
PKC: Protein Kinase C
ROS: Reactive Oxygen Species
